# A pathways-based prediction model for classifying breast cancer subtypes

**DOI:** 10.18632/oncotarget.18544

**Published:** 2017-06-17

**Authors:** Tong Wu, Yunfeng Wang, Ronghui Jiang, Xinliang Lu, Jiawei Tian

**Affiliations:** ^1^ Department of Ultrasound, The Second Affiliated Hospital of Harbin Medical University, Heilongjiang Province, China; ^2^ College of Bioinformatics Science and Technology, Harbin Medical University, Heilongjiang Province, China; ^3^ Department of Surgery, Yanbian No.2 People's Hospital, Jilin Province, China; ^4^ Institute of Immunology, Zhejiang University School of Medicine, Zhejiang Province, China

**Keywords:** breast cancer, subtype-specific gene, pathway enrichment, co-expression network, classification prediction model

## Abstract

Breast cancer is highly heterogeneous and is classified into four subtypes characterized by specific biological traits, treatment responses, and clinical prognoses. We performed a systemic analysis of 698 breast cancer patient samples from The Cancer Genome Atlas project database. We identified 136 breast cancer genes differentially expressed among the four subtypes. Based on unsupervised clustering analysis, these 136 core genes efficiently categorized breast cancer patients into the appropriate subtypes. Functional enrichment based on Kyoto Encyclopedia of Genes and Genomes analysis identified six functional pathways regulated by these genes: JAK-STAT signaling, basal cell carcinoma, inflammatory mediator regulation of TRP channels, non-small cell lung cancer, glutamatergic synapse, and amyotrophic lateral sclerosis. Three support vector machine (SVM) classification models based on the identified pathways effectively classified different breast cancer subtypes, suggesting that breast cancer subtype-specific risk assessment based on disease pathways could be a potentially valuable approach. Our analysis not only provides insight into breast cancer subtype-specific mechanisms, but also may improve the accuracy of SVM classification models.

## INTRODUCTION

Breast cancer is one of the most common cancers among women, with more than 1,300,000 new cases diagnosed, and 450,000 deaths occurring annually worldwide [1]. Clinically, breast cancer is grouped into luminal A (LA), luminal B (LB), human epidermal growth factor receptor 2-positive (HER2+), and triple-negative (TN) subtypes according to estrogen receptor (ER), progesterone receptor (PR), and epidermal growth factor receptor ErbB2/HER2 (HER2) expression. Expression of these receptors is routinely used to select treatments for breast cancer patients and predict prognosis [2]. Breast cancer molecular and behavioral heterogeneity requires the application of different therapeutic methods for each subtype [1, 3, 4]. Despite recent treatment advances, this aggressive disease is still associated with a poor 5-year survival rate [3, 5].

Breast cancer comprises different tumor subtypes, rather than a single cancer type, as characterized by different sets of responsible genes and regulatory pathways [6]. Identification of subtype-specific genes is urgently needed to better understand pathological mechanisms, for improved patient diagnostic and prognostic accuracy, and for a better-refined personalized medicine framework [7, 8]. However, due in part to a lack of access to candidate patients with different tumor subtypes, our current understanding of subtype-specific disease mechanisms is incomplete. However, The Cancer Genome Atlas (TCGA) project provides a catalog of breast cancer and matched normal sample genomic sequencing datasets [1], enabling both identification of subtype-specific genes and corresponding functional analyses [9]. A recent study of breast cancer and glioblastoma multiforme datasets provided by TCGA identified cancer subtype-associated genes using microRNA (miRNA), transcription factor (TF), and messenger RNA (mRNA) expression data and network information [10]. Another study analyzed a highly heterogeneous group of 165 TN breast cancers to determine the main functions of subtype-specific genes and pathways [11]. Yang, *et al*. used next-generation sequencing and bioinformatics techniques to perform an expression profile analysis of long non-coding RNAs (lncRNAs) in the HER-2-enriched subtype [12]. This work provided useful information for exploring candidate therapeutic targets and new molecular biomarkers for this subtype.

In this work, we performed a systemic analysis of 698 TCGA breast cancer patients. Large-scale co-expression analysis was performed for gene pairs in the four different subtypes, and subtype-associated co-expression networks were generated to reflect the specific topological properties. We identified 136 breast cancer genes differentially expressed among the four subtypes. Based on unsupervised clustering analysis, these 136 core genes efficiently categorized breast cancer patients into the appropriate subtypes. In addition, functional enrichment analysis identified six biological pathways associated with these 136 core genes. We analyzed gene co-expression patterns to infer associated pathways and to evaluate dynamic pathway alterations in the different subtypes based on the sliding window [13] and loess fitting [14] methods. Ultimately, we used the six pathways as features to build a support vector machine (SVM) model. Receiver operating characteristic (ROC) curves based on cross-validation indicated that using the mutating pathway feature effectively distinguished the different subtypes. Overall, our analysis not only provided insight into breast cancer subtype-specific mechanisms, but may also improve the accuracy of SVM classification models. The subtype-specific pathways identified here likely include subtype-specific biomarkers and personalized drug targets that warrant further study.

## RESULTS

### Identification of subtype-specific genes

**Figure 1:**
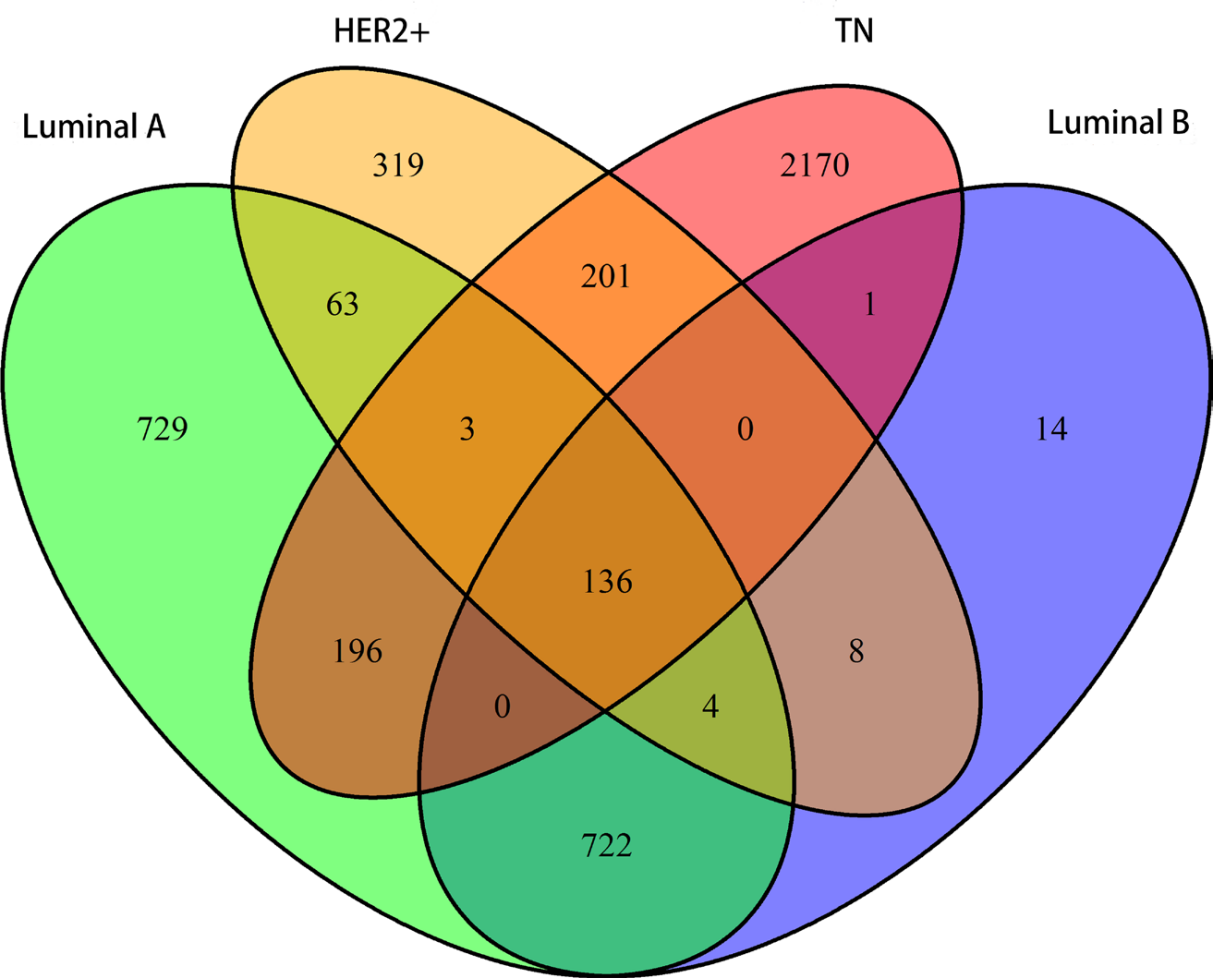
Venn diagram showing overlapping and unique subtype-specific genes Green: LA breast cancer subtype; blue: LB; orange: HER2+; red: TN. 136 genes overlapped between the four specific gene sets. LA, luminal A; LB, luminal B; HER2+, human epidermal growth factor receptor 2 positive; TN, triple negative.

Analysis of variance (ANOVA) was first used to identify differentially expressed genes among the four subtypes. Specific genes for each subtype were further screened using Student's *t* test. Overall, 1853, 885, 734, and 2707 genes were found for the LA, LB, HER2+, and TN subtypes. Although overlap existed, 729, 14, 319, and 2170 subtype-specific genes were identified for the LA, LB, HER2+, and TN subtypes, respectively (Figure 1). Compared with other subtypes, the LA and TN subtypes included more unique genes, suggesting that these genes only exhibited differential expression in these two subtypes. LA and TN represent two extremes among the four breast cancer subtypes, with the former having the lowest degree of malignancy and the latter the highest, which may explain this result.

We also observed differential expression in 136 genes among the four subtypes, indicating that dynamic changes occurred in these genes with increasing malignancy. These genes exhibited a consistent gradient in expression variability, associated with degree of tumor malignancy. Co-expression correlations among these 136 core genes might represent important markers for the different subtypes.

### Correlation analysis

**Figure 2:**
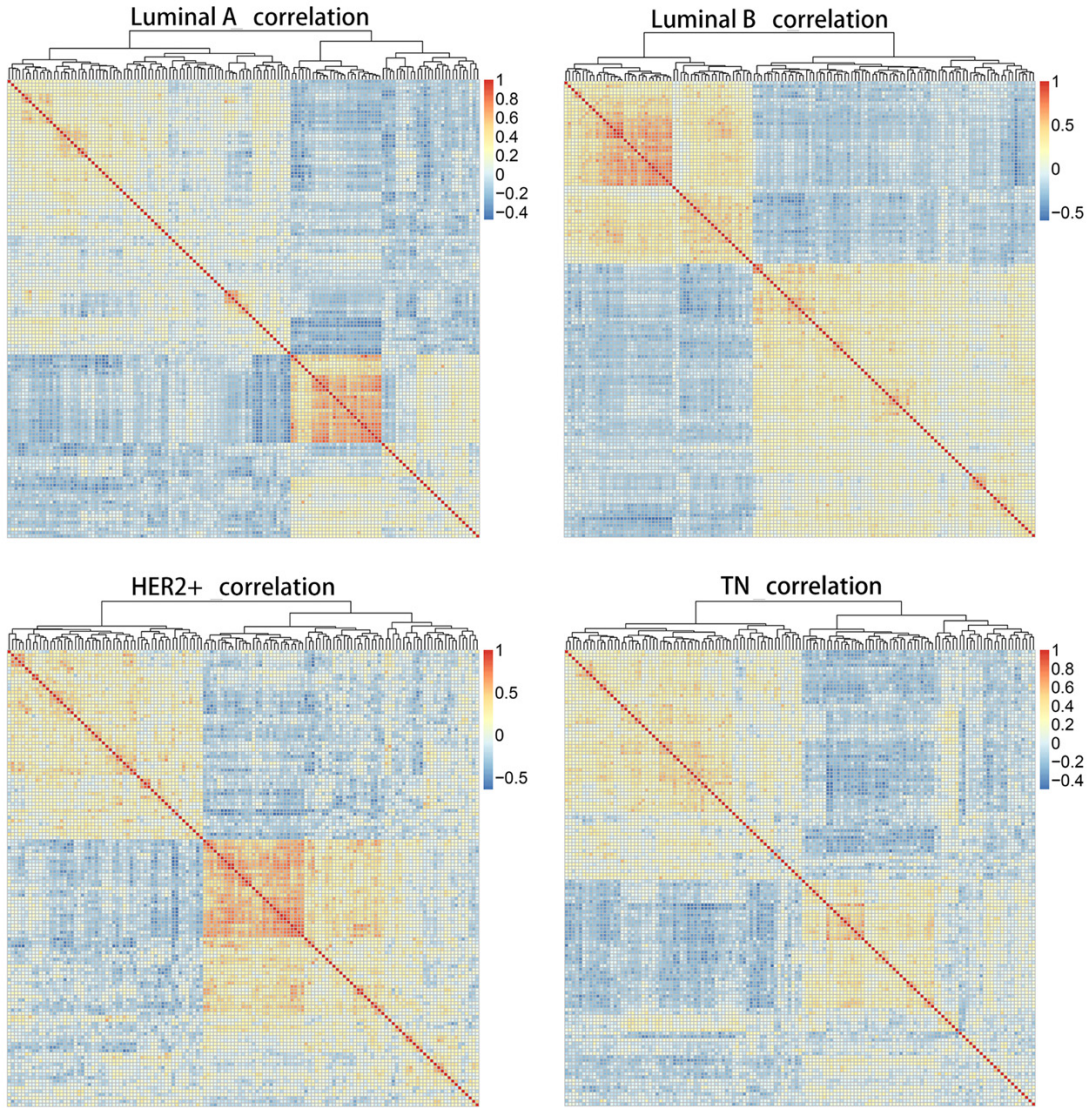
Heat map matrices showing co-expression correlations between 136 overlapping genes for samples of all four subtypes Red: positive correlation; blue: negative correlation. Results indicated that correlations among these 136 genes in each subtype were not identical. HER2+, human epidermal growth factor receptor 2 positive; TN, triple negative.

The Pearson correlation coefficient was used to evaluate correlations among core genes in each of the four subtypes. A correlation coefficient of R > 0.5 indicated a positive correlation, whereas R < −0.5 indicated a negative correlation. Figure 2 shows matrices for the 136 overlapping genes from all four subtypes depicted as heat maps. Correlations among these 136 genes in each subtype were not identical, indicating that gene expression varied between subtypes, and that gene correlations also changed accordingly. These dynamic changes made it possible to distinguish different subtypes at the molecular level.

**Figure 3:**
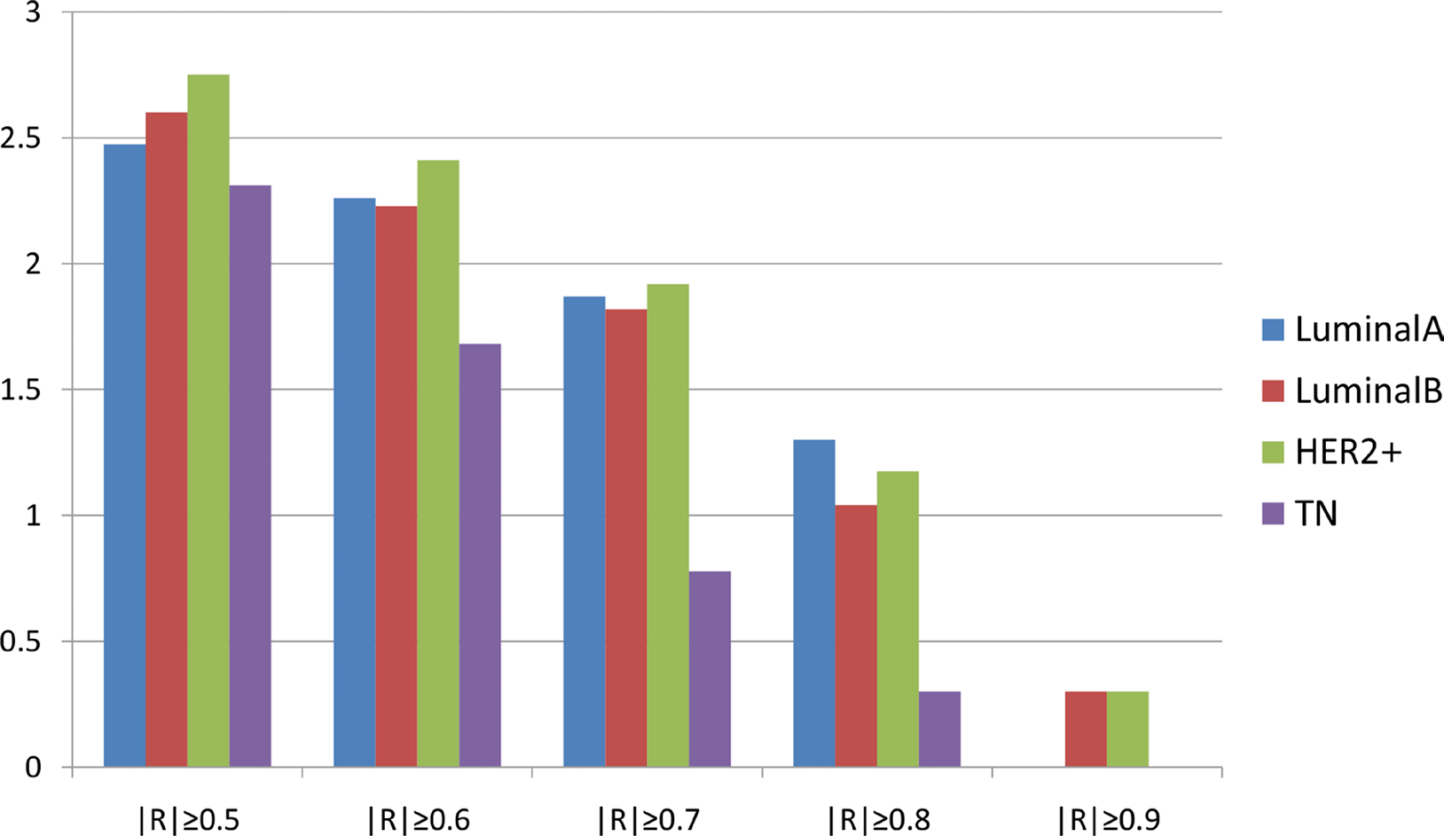
Correlation pairs according to the correlation coefficient Horizontal axis represents the correlation coefficient; vertical axis represents the number of correlated gene pairs after logarithmic conversion. Blue: LA breast cancer subtype; red: LB; green: HER2+; purple: TN. Overall, the number of gene pairs gradually decreased with an increasing R value. LA, luminal A; LB, luminal B; HER2+, human epidermal growth factor receptor 2 positive; TN, triple negative.

We also investigated variation in the number of correlated gene pairs in the four subtypes by increasing the threshold value of R (Table 1). A logarithmic conversion was then performed for the number of correlated gene pairs in the four subtypes with an increasing threshold value of R. Overall, the number of gene pairs gradually decreased with an increasing R value (Figure 3). For each of the correlation coefficients, the number of correlated gene pairs tended to be lower in TN, suggesting that the original correlated gene pairs changed with regard to expression and function. This resulted in the loss of genetic correlation in TN patients.

**Table 1:** Correlation of gene pairs

Group	|R| ≥ 0.5	|R| ≥ 0.6	|R| ≥ 0.7	|R| ≥ 0.8	|R| ≥ 0.9
Luminal A	296	181	73	19	0
Luminal B	398	168	65	10	1
HER2+	562	257	82	14	1
TN	204	47	5	1	0

LA, luminal A; LB, luminal B; HER2+, human epidermal growth factor receptor 2 positive; TN, triple negative.

**Figure 4:**
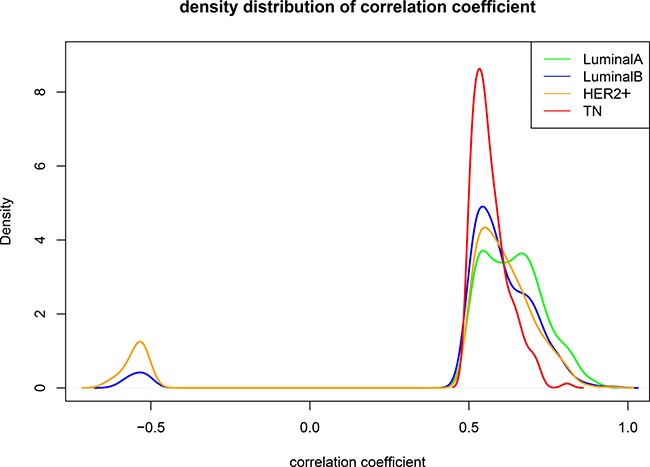
Comparison between subtypes when R ≥ 0.5 Green: LA breast cancer subtype; blue: LB; orange: HER2+; red: TN. Horizontal axis represents the correlation coefficient; vertical axis represents the density distribution. Differences were observed between the density distributions of the correlation coefficients for the four subtypes. LA, luminal A; LB, luminal B; HER2+, human epidermal growth factor receptor 2 positive; TN, triple negative.

Probability density distributions and relationships between overlapping and unique gene pairs were analyzed at R ≥ 0.5 for the correlated gene pairs of the four subtypes. All relevant genes for the LA subtype were positively correlated, and had the highest correlation among the four subtypes (Figure 4). The density began to shift negatively starting with the LB and HER2+subtypes, and negatively correlated gene pairs were found. Compared with the other three subtypes, the number of correlated TN gene pairs was reduced and convergent toward R = 0.5. This suggests that in the transition from LA to TN, correlations may be unstable or lost between gene pairs due to expression variations. Thus, co-expression among genes is an important marker for the different breast cancer subtypes.

### Construction of the co-expression network

**Figure 5:**
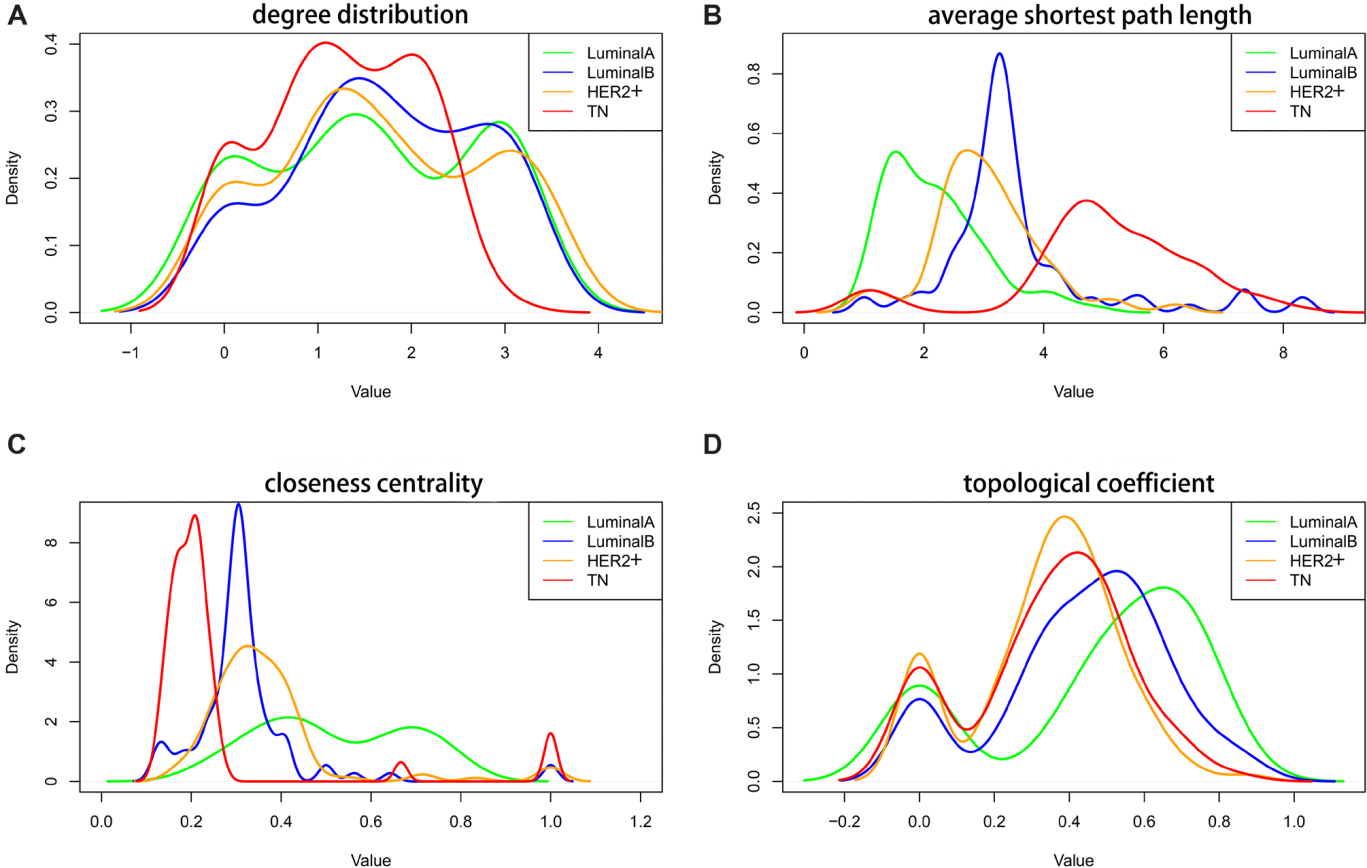
Topological characteristics in the four subtype co-expression networks Degree distribution (**A**), average shortest path length (**B**), closeness centrality (**C**), and topological coefficient (**D**) of the four subtypes. Green: LA breast cancer subtype; blue: LB; orange: HER2+; red: TN. LA, luminal A; LB, luminal B; HER2+, human epidermal growth factor receptor 2 positive; TN, triple negative.

The co-expression network was constructed using genes associated with the four subtypes. Four topological characteristics (degree distribution, average shortest path length, closeness centrality, and topological coefficient) were compared in the co-expression networks. Compared with the other subtypes, the TN subtype peak (red, Figure 5A) shifted to a lower degree of distribution, suggesting that edge deletion occurred in the system network and that the original co-expressed genes gradually lost co-expression during transition to the TN subtype. In the LA to TN subtype transition, average shortest path length peak values gradually shifted to the right, suggesting that the average shortest path gradually increased in the networks from LA to TN and that the network signal transmission efficiency decreased (Figure 5B). The closeness centrality tended to be smaller and more convergent from the LA to TN subtype. Moreover, a small set of genes with large closeness centrality was observed for TN and LB, which suggests an interaction transformation among the genes (Figure 5C). From LA to TN subtypes, the topological coefficient gradually decreased, and the distribution of peak values was lower, suggesting that the system network efficacy decreased over this transition (Figure 5D).

To evaluate the network centrality of the 136 core genes in the four subtype-specific networks, we compared the mean degree of the 136 core genes with all the genes in the four networks (Table 2). Base 10 logarithmic conversion was performed for all nodes. All 136 core genes in the four networks showed a higher distribution than average, suggesting network centrality for these genes in all subtypes and indicating that these genes are important hub nodes.

**Table 2:** Mean and standard deviation of node degree of 136 core genes and all the genes in four subtype-specific networks

Group	Luminal A	Luminal B	HER2+	TN
Average of 136 core genes	1.455246	1.329812	1.278754	1.240074
Mean degree	1.0603975	0.9911572	1.0298602	0.8021931
Standard deviation	0.5863981	0.5515769	0.3697134	0.4997064

HER2+, human epidermal growth factor receptor 2 positive; TN, triple negative.

### Unsupervised hierarchical clustering

**Figure 6:**
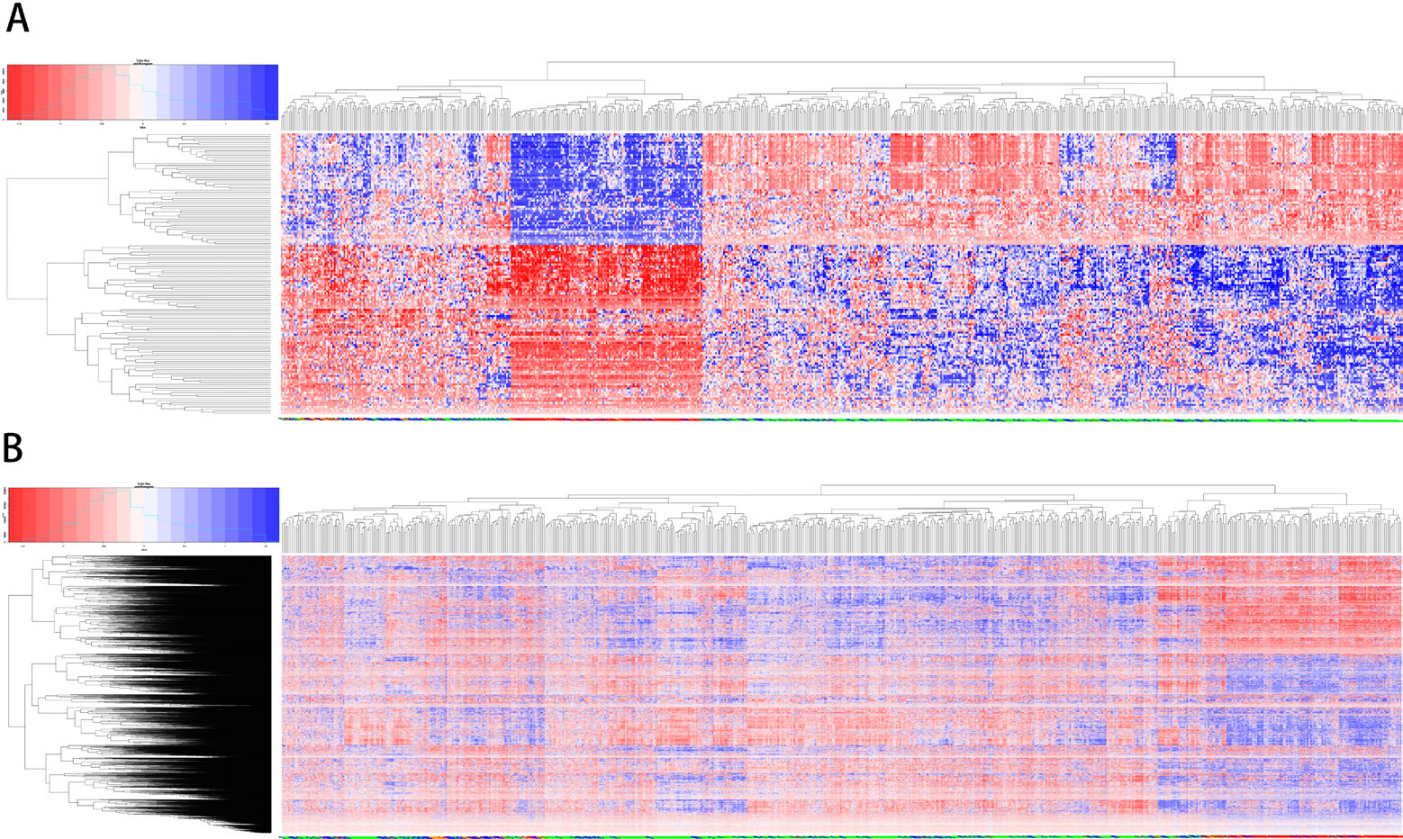
Unsupervised clustering analysis using overlapping and unifying genes The results of unsupervised clustering for samples of all four subtypes using 136 overlapping genes (**A**) and unifying genes (**B**) Red: high expression genes; blue: low expression.

Figure 6 shows the results of unsupervised clustering using intersection (136) and set (3837) genes for samples of all four subtypes. Comparison of the two clustering results showed that the intersection genes clustered better than the set genes. All of the TN cases were concentrated in the same cluster, and most of the LA cases were in another cluster (Figure 6A). TN cases are also separated from the other subtypes when more genes were used (Figure 6B). This confirmed that the 136 core overlapping genes could replace the set genes with similar classification efficiency.

### Functional pathway analysis

Functional enrichment based on Kyoto Encyclopedia of Genes and Genomes (KEGG) analysis was performed for the core genes using Fisher's exact test [15]. Six functional pathways regulated by core genes were identified at a threshold of *P* < 0.05 (Table 3). The JAK/STAT pathway, a functional pathway involved in cell cycle and apoptosis regulation, was identified in the enrichment analysis, and JAK/STAT-mediated PI3K-Akt signaling has been correlated with drug resistance in breast cancer [16]. Detection of the inflammatory mediator regulation pathway suggests that oxidative stress and immune response are both important, although their functional levels may differ in distinct subtypes. Multiple studies have associated glutamic acid with breast cancer prognosis [17]. One disease pathway, amyotrophic lateral sclerosis (ALS), was also enriched. Although there is insufficient evidence to show a relationship between ALS and breast cancer, glutamic acid accumulation in nerve cells is one of the main pathogenic factors for ALS [18]. Two canonical cancer-related pathways, basal cell carcinoma and non-small cell lung cancer, were also enriched. In addition to the functional enrichment of core genes, we also performed a functional analysis of the genes specific for each subtype (Supplementary Material).

**Table 3:** Functional pathway enrichment analysis of 136 core genes

Pathway	Count	*P*-value
JAK-STAT signaling pathway	12	1.65E-04
Basal cell carcinoma	18	2.94E-04
Inflammatory mediator regulation of TRP channels	22	0.009909
Non-small cell lung cancer	14	0.020347
Glutamatergic synapse	10	0.040388
Amyotrophic lateral sclerosis (ALS)	6	0.049967

### Pathway alteration scores

**Figure 7:**
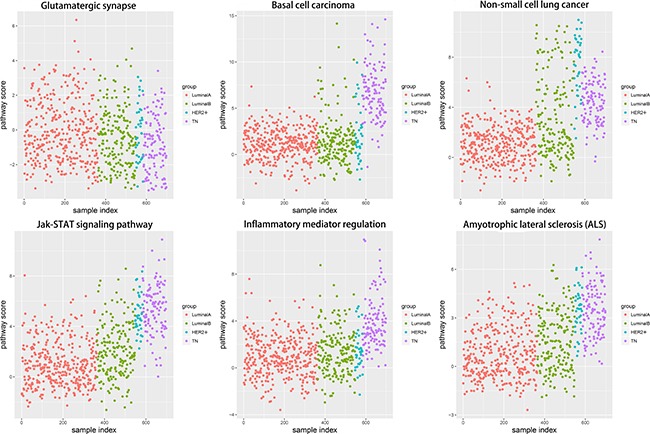
Alteration score distributions for six functional pathways in the four breast cancer subtypes Diffused points show the distribution of alteration scores of the functional pathways. The six functional pathways included glutamatergic synapse, basal cell carcinoma, non-small cell lung cancer, JAK-STAT signaling pathway, inflammatory mediator regulation of TRP channels, and amyotrophic lateral sclerosis. Red: LA breast cancer subtype; green: LB; blue: HER2+; purple: TN. LA, luminal A; LB, luminal B; HER2+, human epidermal growth factor receptor 2 positive; TN, triple negative.

Corresponding gene expression values were used to calculate alteration scores for the six pathways to determine the distributions of their variations in the different breast cancer subtypes (Figure 7). LA subtype cases fluctuated near zero and were close to the normal state, whereas the other subtypes showed deviations. Notably, the directions of variation in the same pathways were not consistent between different subtypes.

**Figure 8:**
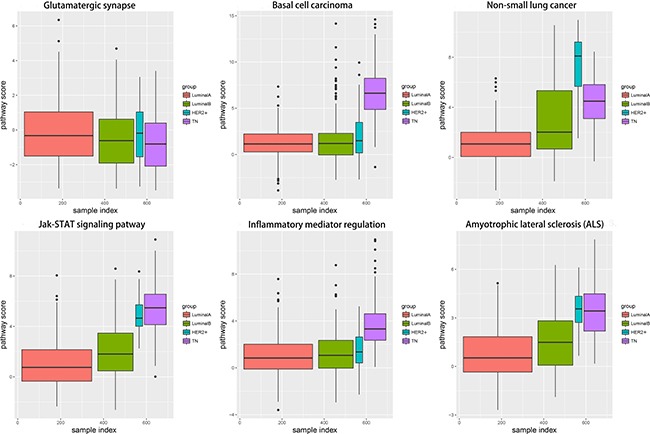
Boxplots showing alteration score distributions for the six functional pathways in the four breast cancer subtypes Horizontal axis represents the subtype samples; vertical axis represents the alteration score; black horizontal line represents the median. Red: LA breast cancer subtype; green: LB; blue: HER2+; purple: TN. LA, luminal A; LB, luminal B; HER2+, human epidermal growth factor receptor 2 positive; TN, triple negative.

Changes in each pathway among the four subtypes tended to be continuous and linear (Figure 8). The functional level in the transition from LA to TN was either continuously increasing or continuously declining, which suggested that a dynamic linear change occurred at the functional levels of these pathways according to increasing degree of breast cancer malignancy. In contrast, pathway variations in the TN subtype were the most obvious; although glutamatergic synapse showed negative variations, all other pathways exhibited positive variations, suggesting that functional levels were enhanced with increased breast cancer malignancy. The increasing levels of these pathways suggest a shift toward increased malignancy and/or inflammation in the cancer cells.

### Sliding window and LOESS fitting

**Figure 9:**
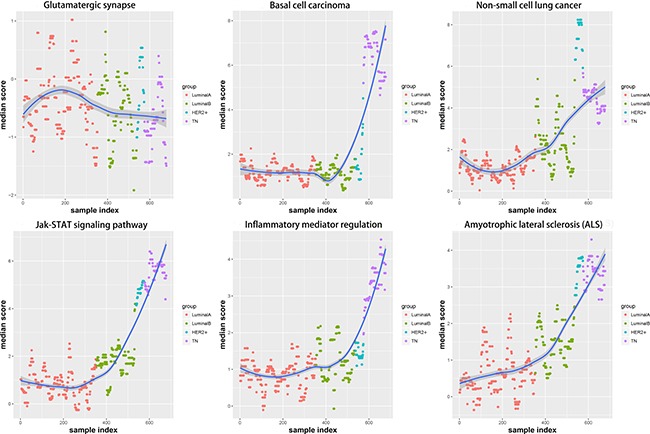
Functional variation trends in the six pathways are shown, using the sliding window approach and LOESS fitting algorithm Red: LA breast cancer subtype; green: LB; blue: HER2+; purple: TN; dark blue: fitting line. LA, luminal A; LB, luminal B; HER2+, human epidermal growth factor receptor 2 positive; TN, triple negative.

To analyze dynamic changes in functional levels with increasing degrees of malignancy in the four subtypes, we applied the sliding window approach [13] to integrate pathway scores for the four subtypes (Figure 9). The LOESS fitting algorithm [14] was used to smooth the data. The pathway functional levels were typically smooth in LA, but either increased or decreased in LB. In TN and HER2+, the pathway functional levels showed continuous change. This phenomenon again confirmed that the LA functional level was most similar to the normal state and that the degree of functional variation was most obvious in TN. Functional variation began to change starting from LB, and the HER2+ functional level was between that of LB and TN. The TN subtype is associated with the poorest prognoses.

### Establishment of the SVM model

**Figure 10:**
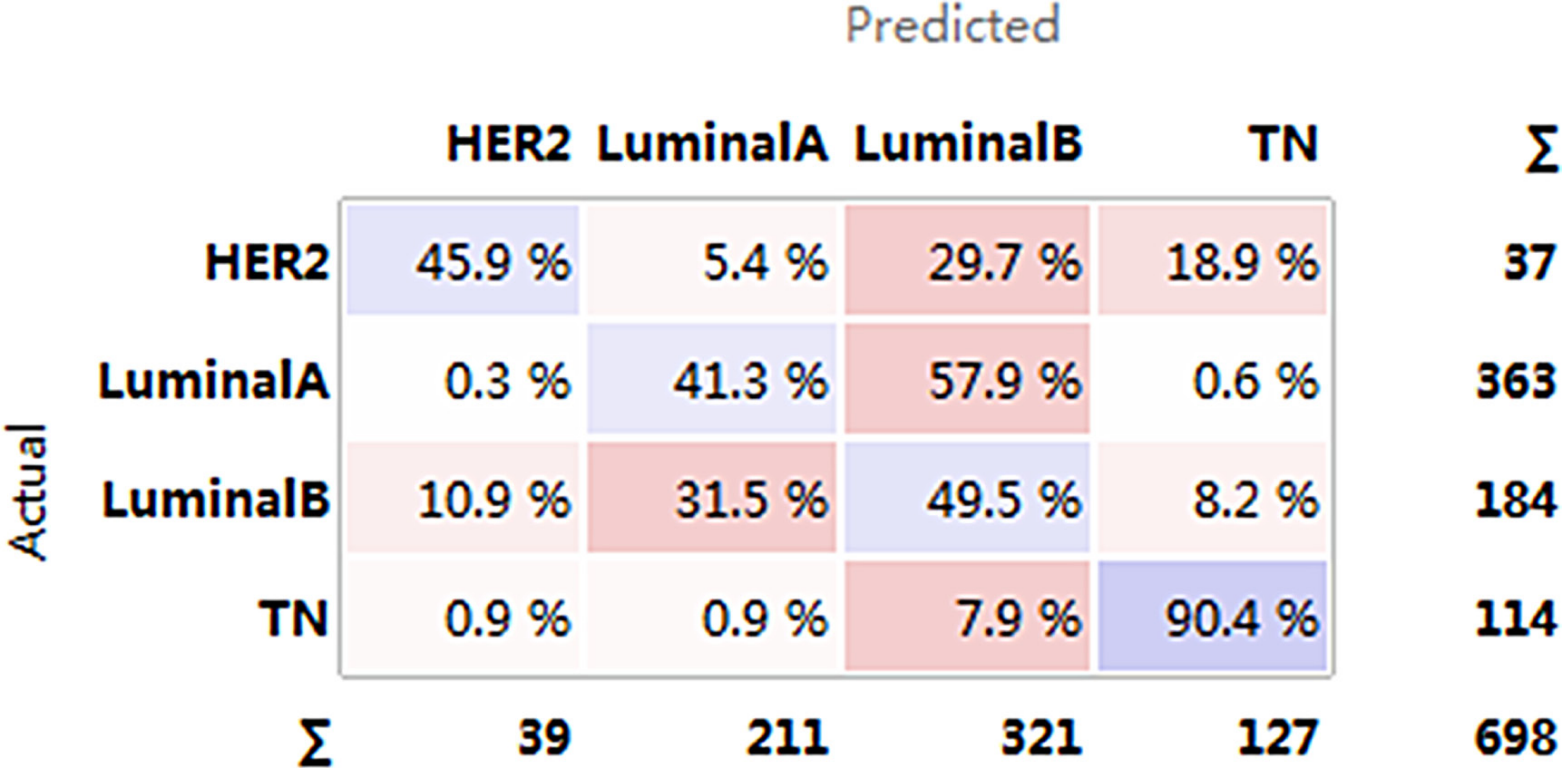
Confusion matrix for the four subtypes Horizontal axis represents the predicted result; vertical axis represents the actual result. Darker color: higher precision; lighter color: lower precision. HER2+, human epidermal growth factor receptor 2 positive; TN, triple negative.

Despite the small sample size for the HER2+ subtype, differences in functional level from the luminal and TN subtypes were observed. Therefore, we attempted to establish an SVM model under an imbalanced training set. Although LA and LB are difficult to distinguish, more than 90% of luminal-type cases could be correctly distinguished from the other two subtypes (Figure 10). Many HER2+ (45.9%) cases were correctly predicted, but prediction failure was observed for the other 54.1%. This low precision was attributed to the small HER2+ patient sample size. 90.4% of TN patients were correctly classified. In addition, three individual models were built for pairwise comparison after excluding the HER2+ patients.

**Figure 11:**
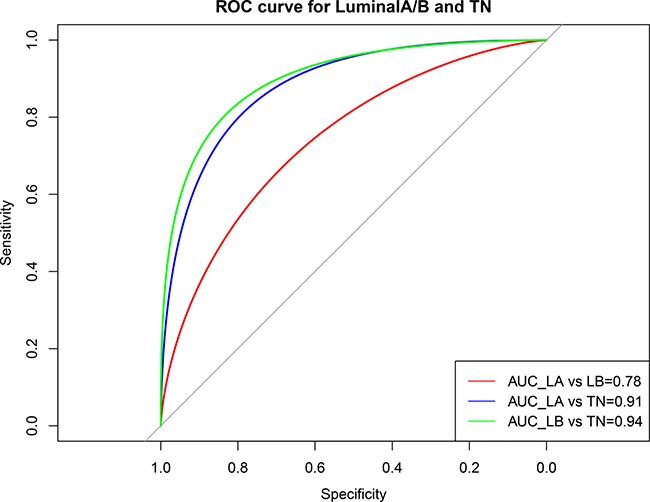
ROC curve showing the performance of the luminal A/B and TN subtype prediction models Horizontal axis represents the ROC curve specificity; vertical axis represents the sensitivity. The average area under the curve (AUC) of the LA and LB was 0.78, but the classification efficiencies for TN and luminal A/B were higher than 91%. Red: AUC for LA and LB; blue: AUC for LA and TN; green: AUC for LB and TN. LA, luminal A; LB, luminal B; TN, triple negative.

A ROC curve analysis was used to assess the performance of the prediction model for the TN and luminal A/B subtypes (Figure 11). The average area under the curve (AUC) of the luminal A and luminal B was 0.78, whereas the classification efficiencies for TN and luminal A/B were > 91%, indicating no obvious functional difference between the different luminal subtypes. However, the TN functional level was altered compared with luminal subtype. These results confirmed that such variable pathways can be used to effectively predict luminal and TN subtypes in breast cancer patients.

The validity of each model was verified by the ROC curves. To comprehensively evaluate the efficacy of the model, the precision, recall, and f1 scores were analyzed. Average precision and recall values for the TN subtype were higher than those for the luminal subtype, but the prediction efficacy of TN alone was inferior to that of the luminal subtype (Table 4). This may have been due to the imbalanced sample sizes of the two groups. Without weighting to adjust for sample size, the average classification efficacy of the model was still > 94%, which again indicated that the six identified variable pathways reflected different functional levels in the different subtypes.

**Table 4:** Classification report for TN/luminal subtypes

Class	Precision	Recall	F1-score	Support
TN	0.76	0.91	0.83	57
Luminal	0.98	0.94	0.96	274
Avg/total	0.94	0.94	0.94	331

TN, triple negative.

## DISCUSSION

Breast cancer is a highly heterogeneous disease with varying biological features and clinical characteristics [19, 20]. Identifying the cancer subtype is important for development of a personalized medicine framework, as correctly classifying tumors increases the likelihood of choosing the most effective patient treatment strategies and appropriately evaluating prognoses [1, 3, 4]. Furthermore, because different subtypes respond to specific treatment modalities, subtype-specific genes involved in the biological processes that confer disease risk must be identified to better classify patients [21, 22].

Information from biological networks, such as co-expression networks, is regularly used with existing computational methods to investigate cancer development and subtype-specific regulatory mechanisms [23]. In the present study, we performed a large-scale co-expression analysis involving 698 TCGA breast cancer patients and built four co-expression networks. We identified breast cancer subtype-specific genes and found 136 core genes that were differentially expressed among the four subtypes. These 136 core genes effectively categorized breast cancer patients into the different subtypes based on unsupervised clustering analysis.

Six biological pathways were identified as associated with these 136 core genes. Notably, our analysis identified the JAK/STAT signaling pathway, which participates in regulating the cell cycle and apoptosis. Some studies have correlated JAK/STAT-mediated PI3K-Akt signaling with drug resistance in different breast cancer subtypes [24]. Inflammatory mediator regulation suggests differences in immunity levels between the breast cancer subtypes, and the proinflammatory response following chemotherapy is often associated with differing chemosensitivity [25]. The involvement of glutamatergic synapse and ALS pathways suggests that glutamate may be a subtype-specific marker. Indeed, glutamatergic signaling inhibition suppresses breast cancer growth, especially in the TN subtype [26]. The other two pathways identified were canonical cancer-related pathways: basal cell carcinoma and non-small cell lung cancer. Lung metastasis is often observed in breast cancer patients, as is bone and liver metastasis, and studies have shown that patients with different subtypes have varying risks of lung metastasis [27, 28].

We evaluated dynamic pathway alterations among the different subtypes using the sliding window [13] and LOESS fitting [14] methods. The classic functional and disease pathways identified in this study showed functional level differences among the four subtypes, exhibiting primarily linear changes correlated with degree of malignancy. Traditional methods of risk assessment use a variety of breast cancer-related genes, although systematic risk assessment for certain subtypes has been unavailable to date. The pathways we identified exhibited differences among the different subtypes, suggesting that breast cancer subtype-specific risk assessment based on disease pathways could be a potentially valuable approach. Finally, the breast cancer subtype-associated pathways used as features to build the SVM model effectively classified luminal and TN subtypes with high accuracy.

In conclusion, our study provides a comprehensive analysis of breast cancer subtype genes across 698 TCGA samples. In addition to individual genes, disease pathways may constitute a valuable subtype-specific breast cancer risk assessment tool. Altered pathways were used as new features to classify different subtypes with high accuracy. Additionally, because these functional pathways exhibited differences between subtypes, they may include specific therapeutic targets, and warrant further analysis. Although our study sample size was somewhat limited and prediction was only performed for the luminal A/B and TN subtypes, our approach may provide new directions for improving breast cancer patient personalized therapies. Our method can be used to accelerate the discovery of molecular biomarkers and, potentially, to more completely characterize the molecular architectures of breast cancer subtypes.

## MATERIALS AND METHODS

### Data acquisition

We used the TCGAbiolinks R package (2.3.16) to acquire breast cancer RNAseq expression data from the TCGA database, including 1212 samples and 20,532 corresponding genes [29]. Based on ER, PR, and HER2 expression, breast cancer cases were classified into one of four clinical subtypes: LA subtype (ER+, PR+, HER2−), HER2+ subtype (ER−, PR−, HER2+), TN subtype (ER−, PR−, HER2−), and LB subtype (ER+, PR−, HER2-| ER+, HER2+). Cases with incomplete ER, PR, and HER2 data were excluded. A total of 698 cases were categorized into subtypes as follows: 363 LA, 184 LB, 37 HER2+, and 114 TN. Expression data were normalized using the z-score [30–33] for three reasons. First, we tended to adjust all genes to the normal distribution (mean = 0, sd = 1), which makes them comparable before subsequent differential analysis. Second, some conserved genes showed very small fluctuations. Although these conserved genes are important disease-related genes, they can be easily omitted because of the slight expression change (sd < 1) due to evolutionary conservation. The z-score is a suitable method for identifying these genes because the signals would be enhanced if biased from the reference, and this method has been widely used in non-invasive prenatal testing (NIPT). Third, the corrected gene values were used to calculate the alteration scores of pathways acting as features in the classification model. As all genes are expected to have the same weight, we adjusted them to the normal distribution using the z-score method.

### Identification of subtype-specific genes

Gene sets with specific expression values were identified for each subtype. ANOVA was performed to extract differential genes for the four subtypes, with a significance threshold of *P* < 0.05. A differentiation test was performed for each of the resultant significant genes using Student's *t*-test. If the *P*-values of gene i in subtype M were all < 0.01 compared with the other subtypes, this gene was assigned as a subtype M-specific gene.

### Correlation analysis

Genes with functional correlations often show co-expression correlations that dynamically change with disease progression or subtype. To investigate dynamic correlation characteristics, Pearson correlation analysis was performed for the core genes of each subtype. Genes specifically associated with each subtype and gene pairs universally associated with all four subtypes were identified. The Pearson threshold was set at R = 0.5.

### Construction of a co-expression network

Co-expression networks were constructed based on associated genes, with the genes associated with each subtype set as nodes. If the correlation coefficient was > 0.5 or < −0.5, an edge between the two nodes was assigned. The topological properties of the network were analyzed using the Cytoscape software to identify the specific topological properties of the four subtypes.

### Hierarchical clustering analysis

The correlation matrix was constructed using correlations between core genes, and clustering of the samples and genes was achieved via unsupervised hierarchical clustering, thereby verifying the efficacy of classifying the four subtypes according to core genes. To demonstrate the efficacy of classifying individual patient samples using the core genes, we unified the four specific gene sets to cluster the samples of the four subtypes by comparison.

### Functional analysis

Core gene expression differences were observed among the four subtypes, suggesting functional differences for core gene-regulated pathways between subtypes. To identify these differentiated functions, KEGG functional enrichment analysis of the core genes was conducted using Fisher's exact test. In addition to core genes, functional analysis of the genes specific for each subtype was also performed. These functions would be used as characteristics to establish independent models and predict any two subtypes.

### Pathway alteration scores

The functional level of pathways in each of the four subtypes was quantified by calculating the alteration score of each pathway as follows:

$$\text{Equation }1:\text{score}\left(\text{P}\right)=\text{logei}\frac{\sqrt{\sum_{i=1}^{m}{\left(Gi1\mu \right)}^{2}}}{\sqrt{\sum_{j=1}^{n}{\left(Gj1\mu \right)}^{2}}})$$

where pathway P contains N enriched genes, m indicates unregulated genes, and n indicates downregulated genes for any gene G. The LA subtype served as the control, and μ is the mean expression of G in LA. A higher score (P) indicated more obvious positive variation of the pathway, whereas a smaller score (P) indicated more obvious negative variation.

### Sliding window and LOESS fitting

The alteration score of the variation pathway was calculated for each sample. To filter out interfering noise signals while observing dynamic changes in the pathway during transition between two subtypes, the score of each pathway was integrated using the sliding window approach [13]. From the starting end, every 20 samples were integrated as a window, with 10 samples overlapping between adjacent windows. The median value for each window was fitted using the LOESS fitting algorithm [14].

### Establishment of the classification model

An unsupervised clustering of core genes distinguished patients with TN breast cancer from those with luminal subtypes. Functional level differences between the subtypes were also verified for the pathways regulated by these genes. Therefore, using the functional pathways as features, cases were classified using a supervised SVM algorithm. Feature values of each pathway were encoded using Equation 1, and we ultimately obtained a matrix consisting of variation pathways and 698 samples. The matrix was normalized using the z-score method. To avoid over fitting, default parameters were used for SVM. Penalty parameter C of the error term was set to 1; kernel type to be used in the algorithm was set to ‘rbf’; degree of polynomial kernel function was set to 3; kernel coefficient gamma was set to 0. Four sample sets were used to train the model, and its classification efficacy was reflected by the confusion matrix. To further distinguish any two subtypes, the following treatments were carried out: (1) Because the number of HER2+ samples was small, they were removed, and only luminal A/B and TN were compared. (2) Pathways enriched in two subtype-specific genes were combined to construct and normalize eigenvalue matrices. (3) Five folds cross-validation was applied; that is, four folds samples were randomly selected for training, and the remaining one was used for the test. This process was repeated five times until all samples were predicted once. (4) Combined with the cross-validation results, a ROC curve was generated to assess classification performance.

## SUPPLEMENTARY MATERIALS TABLES










