# Supplementary material for: A pathways-based prediction model for classifying breast cancer subtypes
Source: Oncotarget. 2017 Jun 17;8(35):58809–22. doi: 10.18632/oncotarget.18544 (PMC5601695; doi:10.18632/oncotarget.18544)
Supplement: Supplementary file 1 [file oncotarget-08-58809-s001.pdf]

A pathways-based prediction model for classifying breast cancer subtypes

Supplementary Materials

Supplementary Table 1: Functional pathway analysis of the genes specific for TN subtype. See Supplementary_Table_1

Supplementary Table 2: Functional pathway analysis of the genes specific for HER2+ subtype. See Supplementary_Table_2

Supplementary Table 3: Functional pathway analysis of the genes specific for LA subtype. See Supplementary_Table_3

Supplementary Table 4: Functional pathway analysis of the genes specific for LB subtype. See Supplementary_Table_4
